# Multiple sclerosis and pregnancy: what does the patient think? a questionnaire study

**DOI:** 10.1186/1756-0500-3-91

**Published:** 2010-04-03

**Authors:** Peter Albrecht, Dorothea Fischer, Andreas Moser

**Affiliations:** 1 Department of Neurology, University of Lübeck, Ratzeburger Allee 160, 23538 Lübeck, Germany; 2 Department of Gynaecology and Obstetrics, University of Lübeck, Ratzeburger Allee 160, 23538 Lübeck, Germany

## Abstract

**Background:**

Multiple Sclerosis (MS) is primarily a disease of women in their childbearing years. Pregnancy and puerperium have opposite effects on the course of the disease. Nevertheless, no studies have been carried out yet on the level of information among female MS-patients regarding the interaction between MS and pregnancy.

**Findings:**

Demographic data, clinical features of MS, course of MS during pregnancy and puerperium as well as knowledge concerning MS and pregnancy were evaluated by means of a questionnaire in 154 female MS-patients. The level of information was significantly higher (p < 0.001) in women who had been pregnant in the past with the diagnosis MS known at this point of time. Furthermore patients reported about a lower frequency of relapses during pregnancy and a higher frequency of relapses in the first six months after giving birth.

**Conclusions:**

The findings illustrate a lack of knowledge in female MS-patients concerning the interactions of MS and pregnancy. In order to make their own independent decision based on scientific facts known to date, female MS-patients need to be better informed on issues regarding MS and pregnancy.

## Introduction

Multiple Sclerosis (MS) is a chronic autoimmune disease of the central nervous system (CNS). It primarily affects premenopausal women [1,2]. Several studies show, that pregnancy in MS-patients is associated with a lower risk of progression and a lower risk of exacerbation, whereas the relapse rate increases during the first three months post partum [3-6]. There is no effect of pregnancy on the lifetime course of the disease and long term disability [4,6]. However, it has not been studied yet, to what extent patients know about the interactions between pregnancy and MS and how patients perceive the course of the disease during and after pregnancy. Thus our aims were twofold: 1) to investigate how patients perceive the course of the disease during and after pregnancy and 2) to evaluate the level of knowledge regarding MS and pregnancy.

## Methods

### Survey sample

Prior to conducting the study, the University of Luebeck Ethics Committee approved the study and the questionnaire. The questionnaire was mailed to 300 women with definitive MS who had been in contact with the Department of Neurology of the University of Luebeck in the past. The study was carried out November 06 - June 07, age of the patients was 18-56 years. Additionally to the questionnaire, the patients were provided a pre-addressed, pre-stamped envelope and were instructed to return their completed questionnaire with no further identifying information.

### Questionnaire design

A questionnaire based survey was designed to gather data on the following in female MS-patients:

1) general demography, course of disease and medical treatment,

2) perceived course of MS during and after pregnancy;

3) knowledge and personal beliefs regarding:

▪ a possible effect of MS on pregnancy

▪ a possible effect of pregnancy on MS

▪ a possible effect of the puerperium on MS

▪ a possible effect of breastfeeding on MS

The questionnaire used was a 7 page 31 item survey, the language was German [Additional file 1]. The questionnaire can be requested from the authors.

### Statistics

Analyses were conducted using statistical software (SPSS vs.14 for windows). Data are presented as summary statistics (mean and median as appropriate). Ordered categorical data were summarized as proportions. Significance was set at the 95% level.

## Results

154 questionnaires were returned (response rate 51.3%). Data from 150 patients could be analyzed. However, since several questions were not answered by all participants of the study, the sample-size varies for single questions.

### General demography, course of disease, medical treatment and walking ability

Mean age was 39.3 years, median 40, SD 9. Of 147 female MS-patients, 90 women (61.2%) experienced a relapsing remitting (RR) course of MS, 20 women (13.6%) a secondary progressive (SP) and 6 women (4.1%) a primary progressive (PP) course of MS. 31 patients (21.1%) were not sure about their course of MS. 92 of 149 patients (61.7%) were treated with an immunomodulatory or immunosuppressive agent.

### Perceived course of MS during and after pregnancy

94 (62.7%) of 150 women had at least one child. In 36 women (24%) the diagnosis MS was known at the time of their last pregnancy. 35 of these women rated the course of MS and the relapse rate during pregnancy. 26 women of this subgroup (74%) experienced a lower relapse rate during pregnancy, whereas nine women (26%) reported about a relapse rate during pregnancy that was as high or higher compared to the same period of time before pregnancy. Of 35 women 21 patients (60%) reported about a higher relapse rate for the first six months after giving birth compared to the period of pregnancy, six patients (17%) experienced no difference in the relapse rate and four patients (11.5%) reported about a lower relapse rate during this period of time. For four women (11.5%) this period of time was not over yet.

### Level of knowledge regarding MS and Pregnancy

**Figure 1:**
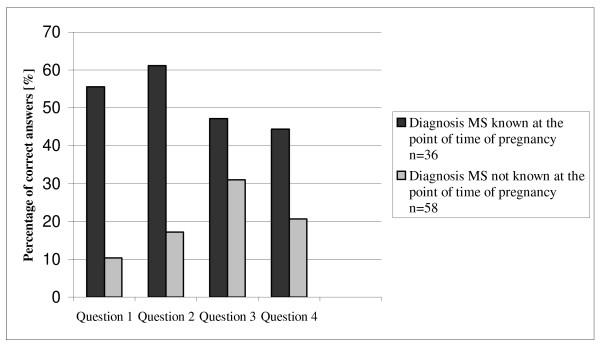
**Percentage of correct answers in female MS-patients who have been pregnant in the past**.

Four questions concerning MS and pregnancy were asked using multiple-choice. Four possible answers to each question were given, one of which was correct. Of a total of 146 patients, only seven patients (4.8%) gave correct answers to all four questions. 17 patients (11.6%) answered 3 questions, and 35 patients (24%) answered 2 questions correctly. One correct answer was given by 38 patients (26%) and 49 patients (33.6%) could give none correct answer. We divided the group of MS-patients who were pregnant at least once (n = 94) in a subgroup of women without the diagnosis MS at the point of time of their last pregnancy (n = 58; 61.7%), and in a subgroup of patients with the definite diagnosis MS at the time of their last pregnancy (n = 36; 38.3%; see (Figure 1). The level of knowledge concerning issues related to MS and pregnancy in the group of patients with the diagnosis MS at the time of their last pregnancy was significantly higher (p < 0.001, chi-square-test).

## Discussion

Based on the patients' answers, the rate of relapses decreases during pregnancy, whereas there is a higher rate of relapses for the first six months after giving birth. These data are consistent with results of former clinical studies, [4-6]. The extension of previous knowledge consists of the fact, that the opposite effects of pregnancy and puerperium are perceived by patients on a subjective level as well. The underlying pathophysiological mechanisms are not fully understood yet. Pregnancy leads to a wide range of different physiological changes, including the immune system [7]. In particular a shift in the cytokine profile of pregnant women, from a Th1 towards Th2 cytokine profile seems to play an important role [8-10]. Other hormonal and immunological changes are under discussion [11,12]. Particularly with regard to possible therapeutic options in the future, these mechanisms are currently in the focus of interest [13-17]. However, our study has limitations, one of which is the sample szize. Of 154 patients, only 35 women have been pregnant in the past with the diagnosis MS known at this point of time. The second aim of our study was, to evaluate the level of information among female MS-patients concerning MS and pregnancy. This level was surprisingly low. Nevertheless, it has to be taken into consideration, that the age range of subjects is broad and that views on reproduction of MS have changed over time. However, there was one subgroup of patients who was significantly better informed. This group consisted of women who met two conditions. First, these women were at least pregnant once before, and second, the diagnosis MS was known at this point of time. Therefore we suggest, that the higher level of knowledge in this group of patients is due to their own experience. In view of these results we believe that although there are various means of information today for patients (Internet, booklets, TV etc.) physicians need to provide actively, specifically and personally information on the interactions of pregnancy and multiple sclerosis to female MS-patients.

## Competing interests

The authors declare that they have no competing interests.

## Authors' contributions

PA conceived, designed and performed the analysis. AM conceived the study, supervised the analysis and revised the manuscript. DF provided additional guidance in formulating the analysis and revising the manuscript. All authors contributed to writing the paper and all authors read and approved the final manuscript.

## Supplementary Material

Additional file 1 **Questionnaire multiple sclerosis and pregnancy**. This file contains the original questionnaire, that was mailed to the female MS-patients. The language is German. Click here for file
