# Supplementary material for: Multiple sclerosis and pregnancy: what does the patient think? a questionnaire study
Source: BMC Res Notes. 2010 Apr 3;3:91. doi: 10.1186/1756-0500-3-91 (PMC2853552; doi:10.1186/1756-0500-3-91)
Supplement: Additional file 1 — Questionnaire multiple sclerosis and pregnancy. This file contains the original questionnaire, that was mailed to the female MS-patients. The language is German. [file 1756-0500-3-91-S1.DOC]

# Fragebogen Multiple Sklerose und Schwangerschaft

**Hinweise zum Ausfüllen:** Bitte nehmen Sie sich etwas Zeit und füllen Sie den Fragebogen sorgfältig aus. Anschließend senden Sie uns bitte den Fragebogen in dem beiliegenden frankierten und adressierten Briefumschlag zurück. Bitte schreiben Sie nicht Ihren Absender auf den Briefumschlag, damit völlige Anonymität gewahrt wird. Vielen Dank.

Teil A

Dieser Teil des Fragebogens ist bitte von jeder Teilnehmerin auszufüllen, unabhängig davon, ob bei Ihnen eine neurologische Erkrankung besteht oder nicht. Bitte nur eine Antwort pro Frage ankreuzen.

1. **Aktuelles Datum** ( Bitte Tag - Monat - Jahr eintragen)
2. **Geburtsdatum** (Bitte nur Geburtsmonat und –Jahr eintragen)
3. **Körpergröße in cm**
4. **Anzahl Geschwister**
   1. Anzahl Brüder
   2. Anzahl Schwestern
5. **Halten Sie eine Befragung dieser Art für sinnvoll?**
   1. Sehr sinnvoll
   2. Eher sinnvoll
   3. Eher sinnlos
   4. Völlig sinnlos
6. **Ist bei Ihnen eine Multiple-Sklerose-Erkrankung bekannt?**
   1. Ja
   2. Nein

Wenn Sie die vorhergehende Frage mit „Nein“ beantwortet haben, sind Sie jetzt fertig mit dem Ausfüllen des Fragebogens. Bitte schicken Sie uns nun den Fragebogen in dem beiliegenden Umschlag zurück. Vielen Dank für Ihre Mitarbeit!

Wenn Sie die vorhergehende Frage mit „Ja“ beantwortet haben, füllen Sie bitte den Fragebogen ab hier weiter aus. Danke.

Teil B

In diesem Teil des Fragebogens geht es um Ihre Multiple Sklerose Erkrankung, sowie um Ihre persönliche Meinung zu einigen Themen aus dem Bereich Multiple Sklerose und Schwangerschaft. Dabei gibt es kein „Richtig“ oder „Falsch“. Bitte nur eine Antwort pro Frage ankreuzen. Vielen Dank.

1. **Welche Verlaufsform der MS-Erkrankung liegt derzeit bei Ihnen vor?**
   1. Schubförmig
   2. Sekundär chronisch progredient (fortschreitend)
   3. Primär chronisch progredient (fortschreitend)
   4. Ich bin mir nicht sicher.
2. **Werden Sie derzeit mit einem dieser Medikamente behandelt?**
   1. Interferon beta 1a 30µg i.m. 1x / Woche (Avonex®)
   2. Interferon beta 1a 22 oder 44 µg s.c. 3x / Woche (Rebif®)
   3. Interferon beta 1b jeden 2.Tag s.c. (Betaferon®)
   4. Glatirameracetat tgl. s.c. (Copaxone®)
   5. Azathioprin (Imurek®)
   6. Novantron (Mitoxantron®)
   7. Ich werde derzeit mit keinem dieser Medikamente behandelt.
3. **Wenn Sie mit einem der oben aufgeführten Medikamente behandelt werden, seit wann?** (Bitte Monat und Jahr eintragen).
4. **Sind Sie durch die Multiple Sklerose Erkrankung in Ihrer Gehfähigkeit eingeschränkt?**
   1. Gehfähigkeit ist überhaupt nicht eingeschränkt.
   2. Gehfähigkeit ist etwas eingeschränkt.
   3. Gehfähigkeit ist eingeschränkt, 10 Minuten gehen ist jedoch

möglich.

- 1. Deutlich eingeschränkt, Gehen in Wohnung und kurze Strecken

ist jedoch möglich.

- 1. Sehr eingeschränkt, nur wenige Schritte sind möglich.
  2. Maximal eingeschränkt, Gehen ist nicht möglich.

1. **Besteht bei Ihnen ein Schwangerschafts- und Kinderwunsch?**
   1. Ja, ich habe aktuell einen Kinderwunsch und möchte jetzt

schwanger werden.

- 1. Ja, ich möchte im Prinzip (weitere) Kinder haben, möchte aber im

Moment nicht schwanger werden.

- 1. Ich weiß noch nicht genau ob ich (weitere) Kinder haben möchte.
  2. Nein, ich möchte eher keine (weiteren) Kinder haben.
  3. Nein, ich möchte auf keinen Fall (weitere) Kinder haben.
  4. Ich bin derzeit schwanger

1. **Beeinflußt die Tatsache, daß Sie an einer Multiplen Sklerose erkrankt sind, Ihre Entscheidung möglicherweise schwanger zu werden?**
   1. Nein, die MS-Erkrankung beeinflußt meine Einstellung bezüglich

einer Schwangerschaft überhaupt nicht.

- 1. Eher nein, die MS-Erkrankung beeinflußt meine Einstellung

bezüglich einer Schwangerschaft kaum.

- 1. Teilweise, die MS-Erkrankung spielt eine Rolle in meiner

Einstellung bezüglich einer Schwangerschaft.

- 1. Ja, die MS-Erkrankung spielt eine sehr große Rolle in meiner

Einstellung bezüglich einer Schwangerschaft.

- 1. Ja, die Erkrankung spielt die entscheidende Rolle in meiner

Einstellung bezüglich einer Schwangerschaft.

1. **Was vermuten Sie, wie wirkt sich eine MS-Erkrankung auf einen Schwangerschaftsverlauf aus?**
   1. Eine MS-Erkrankung wirkt sich eher günstig auf einen

Schwangerschaftsverlauf aus.

- 1. Eine MS-Erkrankung wirkt sich eher ungünstig auf einen

Schwangerschaftsverlauf aus.

- 1. Eine MS-Erkrankung hat keinen Einfluß auf einen

Schwangerschaftsverlauf.

- 1. Dazu habe ich keine Vermutung.

1. **Was vermuten Sie, wie wirkt sich eine Schwangerschaft auf eine MS-Erkrankung aus?**
   1. Eine Schwangerschaft wirkt sich eher ungünstig auf eine MS-

Erkrankung auf, es treten z.B. mehr Schübe auf.

- 1. Eine Schwangerschaft wirkt sich eher günstig auf eine MS-

Erkrankung auf, es treten z.B. weniger Schübe auf.

- 1. Eine Schwangerschaft hat keinen Einfluß auf eine MS-

Erkrankung.

- 1. Dazu habe ich keine Vermutung.

1. **Was vermuten Sie, wie wirken sich die ersten drei Monate nach einer Geburt auf den Verlauf einer MS-Erkrankung bei der Mutter aus?**
   1. Eher ungünstig, es treten z. B. mehr Schübe auf.
   2. Eher günstig, es treten z.B. weniger Schübe auf.
   3. Die ersten drei Monate nach einer Geburt haben keine

Auswirkung auf den Verlauf einer MS-Erkrankung.

- 1. Dazu habe ich keine Vermutung.

1. **Was vermuten Sie, wie wirkt sich Stillen auf den Verlauf einer MS-Erkrankung aus?**
   1. Eher ungünstig, es treten z. B. mehr Schübe auf.
   2. Eher günstig, es treten z.B. weniger Schübe auf.
   3. Stillen hat keine Auswirkung auf eine MS-Erkrankung.
   4. Dazu habe ich keine Vermutung.
2. **Was vermuten Sie, besteht für Kinder, bei denen ein Elternteil an einer MS erkrankt ist, eine erhöhte Wahrscheinlichkeit, selbst einmal an einer MS zu erkranken?**
   1. Nein, diese Wahrscheinlichkeit ist nicht erhöht.
   2. Ja, diese Wahrscheinlichkeit ist etwas erhöht.
   3. Ja, diese Wahrscheinlichkeit ist stark erhöht.
   4. Dazu habe ich keine Vermutung.
3. **Was vermuten Sie, könnte eine MS-Erkrankung eines Elternteils in der Zukunft die Versorgung eines Kindes erschweren?**
   1. Nein, da habe ich keine Bedenken.
   2. Vielleicht.
   3. Ja, das vermute ich schon.
   4. Dazu habe ich keine Vermutung.
4. **Haben Sie Kinder?**
   1. Ja
   2. Nein

Wenn Sie diese Frage mit **Nein** beantwortet haben, füllen Sie bitte den Fragebogen weiter ab **Frage 21** aus. Danke! Wenn Sie diese Frage mit **Ja** beantwortet haben, füllen Sie bitte den Fragebogen weiter ab der nächsten **Frage** **Nr. 20** aus. Danke!

1. **War die MS-Erkrankung bei der Geburt Ihres Kindes / Ihrer Kinder bekannt?**
   1. Ja
   2. Nein
2. **Sind Sie derzeit schwanger?**
   1. Ja
   2. Nein

Bitte füllen Sie den Fragebogen ab hier nur weiter aus, falls Sie eine der beiden letzten Fragen, also Nr. 20 oder Nr. 21 mit Ja beantwortet haben. In dem Fall fahren Sie bitte mit Teil C, Frage 22 fort. Vielen Dank. Falls Sie keine der letzten beiden Fragen (Nr. 20 und 21) mit Ja beantwortet haben, sind Sie jetzt fertig mit dem Ausfüllen des Fragebogens. In diesem Fall bedanken wir uns noch einmal ganz herzlich für Ihre Mitarbeit und bitten Sie, den Fragebogen in dem dafür vorgesehenen Briefumschlag an uns ohne Absender zurück zu schicken. Vielen Dank!

# Teil C

1. **Wie viele Kinder haben Sie?**
   1. Ich habe noch kein Kind, bin derzeit schwanger.
   2. Ich habe ein Kind.
   3. Ich habe zwei Kinder.
   4. Ich habe drei Kinder.
   5. Ich habe vier Kinder.
   6. Ich habe fünf Kinder.
2. **Wie alt ist (sind) Ihr (e) Kind(er)?** Bitte Alter in Jahren eintragen.
   1. Ich habe noch kein Kind, bin derzeit schwanger.
   2. Alter des ersten Kindes: Jahre
   3. Alter des zweiten Kindes: Jahre
   4. Alter des dritten Kindes: Jahre
   5. Alter des vierten Kindes: Jahre
   6. Alter des fünften Kindes: Jahre
3. **Sind Sie mit einem der folgenden Medikamente vor Ihrer aktuellen / letzten Schwangerschaft behandelt worden?**
   1. Interferon beta 1a 30µg i.m. 1x / Woche (Avonex®)
   2. Interferon beta 1a 22 oder 44 µg s.c. 3x / Woche (Rebif®)
   3. Interferon beta 1b jeden 2.Tag s.c. (Betaferon®)
   4. Glatirameracetat tgl. s.c. (Copaxone®)
   5. Azathioprin (Imurek®)
   6. Novantron (Mitoxantron®)
   7. Mit keinem dieser Medikamente
   8. Ich bin mit keinem dieser Medikamente behandelt worden, weil

vor meiner Schwangerschaft keine MS bekannt war.

Hinweis: Wenn Sie mit keinem dieser Medikamente behandelt wurden, können Sie die zwei folgenden Fragen überspringen.

1. **Wenn Sie mit einem dieser Medikamente behandelt wurden, wie lange wurden Sie mit diesem Medikament behandelt?** Bitte Monate eintragen.
   1. Monate
2. **Wann wurde die Behandlung mit diesem Medikament, bezogen auf Ihre letzte / aktuelle Schwangerschaft, abgesetzt?** Bitte ankreuzen und ggf. Monate eintragen.
   1. Das Medikament wurde ca. Monate vor Beginn der

Schwangerschaft abgesetzt.

- 1. Das Medikament wurde im Schwangerschaftsmonat

abgesetzt.

- 1. Das Medikament wurde während der Schwangerschaft nicht

abgesetzt.

1. **Wie häufig traten Ihrer Einschätzung nach Schübe der MS-Erkrankung während der aktuellen / letzten Schwangerschaft auf?**
   1. Es traten ungefähr so häufig Schübe auf, wie in der Zeit vor der

Schwangerschaft.

- 1. Es traten eher mehr Schübe auf, im Vergleich zu der Zeit vor der

Schwangerschaft.

- 1. Es traten eher weniger (oder keine) Schübe auf, im Vergleich zu

der Zeit vor der Schwangerschaft.

1. **Wie häufig haben Sie sich während Ihrer Schwangerschaft bei einem Neurologen oder in einer neurologischen Klinik wegen der MS-Erkrankung vorgestellt?**
   1. Ungefähr so häufig wie sonst auch.
   2. Eher häufiger als sonst.
   3. Eher seltener als sonst.
2. **Wie war Ihrer Einschätzung nach während der ersten sechs Monate nach Ihrer Schwangerschaft der Verlauf der MS-Erkrankung im Vergleich zur Schwangerschaft?**
   1. Ich habe keinen Unterschied bemerkt im Vergleich zur

Schwangerschaft.

- 1. Die MS-Symptome haben in den ersten sechs Monaten nach der

Schwangerschaft eher zugenommen.

- 1. Die MS-Symptome haben in den ersten sechs Monaten nach der

Schwangerschaft eher abgenommen.

- 1. Das kann ich nicht beurteilen, weil dieser Zeitraum noch nicht

vorüber ist.

1. **Wie häufig traten Ihrer Einschätzung nach Schübe der MS-Erkrankung während der ersten sechs Monate nach der Schwangerschaft auf?**
   1. Es traten ungefähr so viele Schübe auf, wie während der

Schwangerschaft.

- 1. Es traten eher mehr Schübe auf, als während der

Schwangerschaft.

- 1. Es traten eher weniger Schübe auf, als während der

Schwangerschaft.

- 1. Das kann ich nicht beurteilen, weil dieser Zeitraum noch nicht

vorüber ist.

1. **Wie häufig haben Sie sich in den ersten sechs Monaten nach Ihrer Schwangerschaft bei einem Neurologen oder in einer neurologischen Klinik wegen der MS-Erkrankung vorgestellt?**
   1. Ungefähr so häufig wie sonst auch.
   2. Eher häufiger als sonst.
   3. Eher seltener als sonst.
   4. Das kann ich nicht beurteilen, weil dieser Zeitraum noch nicht

vorüber ist.

Sie sind jetzt fertig mit dem Ausfüllen des Fragebogens. Wir möchten uns an dieser Stelle noch einmal herzlich für Ihre Mitarbeit bedanken. Bitte schicken Sie uns den Fragebogen nun in dem beiliegenden Umschlag ohne Absender zurück. Vielen Dank.
